# Flavonoids with Gastroprotective Activity

**DOI:** 10.3390/molecules14030979

**Published:** 2009-03-03

**Authors:** Kelly Samara de Lira Mota, Guilherme Eduardo Nunes Dias, Meri Emili Ferreira Pinto, Ânderson Luiz-Ferreira, Alba Regina Monteiro Souza-Brito, Clélia Akiko Hiruma-Lima, José Maria Barbosa-Filho, Leônia Maria Batista

**Affiliations:** 1 Laboratório de Tecnologia Farmacêutica Prof. Delby Fernandes de Medeiros – LTF, Universidade Federal da Paraíba - UFPB, Cx. Postal 5009, 58051-970, João Pessoa, PB, Brazil; E-mails: kellylira@gmail.com (K-L.M.); guilherme1_2005@hotmail.com (G-N.D.); meriemily@hotmail.com (M-F.P.); jbarbosa@ltf.ufpb.br (J-M.B-F.); 2 Laboratório de Produtos Naturais, Universidade Estadual de Campinas - UNICAMP, Cx. Postal 6109, 13083-970, Campinas, SP, Brazil; E-mail: domcarecone@yahoo.com.br (A.L-F.); abrito@unicamp.br (A-M.S-B.); 3 Departamento de Fisiologia, Instituto de Biosciência, São Paulo, Universidade Estadual de São Paulo-UNESP, c.p. 510, Zip Code: 18618-000, Botucatu, SP, Brazil; E-mail: hiruma@ibb.unesp.br (C-A.H-L.)

**Keywords:** Flavonoids, Gastroprotective activity, Peptic ulcers, Natural products.

## Abstract

Peptic ulcers are a common disorder of the entire gastrointestinal tract that occurs mainly in the stomach and the proximal duodenum. This disease is multifactorial and its treatment faces great difficulties due to the limited effectiveness and severe side effects of the currently available drugs. The use of natural products for the prevention and treatment of different pathologies is continuously expanding throughout the world. This is particularly true with regards to flavonoids, which represent a highly diverse class of secondary metabolites with potentially beneficial human health effects that is widely distributed in the plant kingdom and currently consumed in large amounts in the diet. They display several pharmacological properties in the gastroprotective area, acting as anti-secretory, cytoprotective and antioxidant agents. Besides their action as gastroprotectives, flavonoids also act in healing of gastric ulcers and additionally these polyphenolic compounds can be new alternatives for suppression or modulation of peptic ulcers associated with *H. pylori.* In this review, we have summarized the literature on ninety-five flavonoids with varying degrees of antiulcerogenic activity, confirming that flavonoids have a therapeutic potential for the more effective treatment of peptic ulcers.

## Introduction

Peptic ulcers are a common disorder of the entire gastrointestinal tract [1]. They occur mainly in the stomach and the proximal duodenum. They can also occur in the esophagus, jejunum and gastric anastamotic site [2]. A peptic ulcer results from an imbalance between some endogenous aggressive factor(s) [hydrochloric acid, pepsin, refluxed bile, leukotrienes, reactive oxygen species (ROS)] and cytoprotective factors, which include the function of the mucus-bicarbonate barrier, surface active phospholipids, prostaglandins (PGs), mucosal blood flow, cell renewal and migration, nonenzymatic and enzymatic antioxidants and some growth factors [3,4,5,6]. The pathogenesis of gastric ulcers remains widespread, it is multifactorial disease where diverse factors such as a stressful lifestyle, alcohol consumption, use of steroidal and nonsteroidal antiinflammatory drugs (NSAIDs) and drugs which stimulate gastric acid and pepsin secretion, *Helicobacter pylori* infections, smoking, lower socio-economic status and family history all represent significant risk factors that may contribute to increasing gastric damage [3]. The prevention or cure of peptic ulcers is one of the most important challenges confronting medicine nowadays, as it is certainly a major human illness affecting nearly 8 to 10 % of the global population [7], and of these 5% suffer from gastric ulcers [3]. The prevalence of this disease is higher in men than in women [8].

Although recent advances in our understanding have highlighted the multifactorial pathogenesis of peptic ulcers, secretion of gastric acid is still recognized as a central component of this disease, therefore the main therapeutic target is the control of this secretion using antacids, H_2_ receptor blockers like ranitidine, famotidine, anticholinergics like pirenzepin, telezipine or proton pump blockers like omeprazole, lansoprazole, *etc*. [9]. However, gastric ulcer therapy faces nowadays a major drawback because most of the drugs currently available in the market show limited efficacy against gastric diseases and are often associated with severe side effects [10,11]. 

In this context, the use of medicinal plants is in continuous expansion all over the world for the prevention and treatment of different pathologies, and natural products are recovering space and importance in the pharmaceutical industry as inspiring sources of new potentially bioactive molecules [12]. Clinical research has confirmed the efficacy of several plants for the treatment of gastroduodenal diseases [13,14]. The medicinal properties of many plants are attributed mainly to the presence of flavonoids, but they may be also influenced by other organic and inorganic compounds such as coumarins, alkaloids, terpenoids, tannins, phenolic acids and antioxidant micronutrients, *e.*g., Cu, Mn, Zn [15,16].

**Figure 1:**
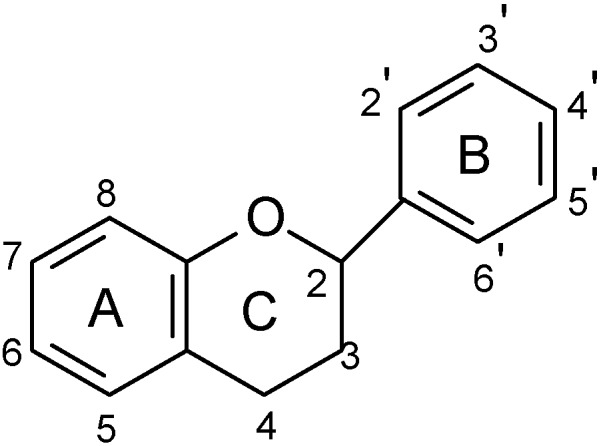
Basic flavonoid structure.

Flavonoids represent a highly diverse class of secondary metabolites comprising about 9,000 structures that have been identified to date. They constitute the largest and most important group of polyphenolic compounds in plants. These compounds are found in all vascular plants as well as in some mosses [17,18]. The term flavonoid is used to describe plant pigments, mostly derived from benzo-γ-pyrone, which is synonymous with chromone (rings A and C in Figure 1) [19,20]. 

**Scheme 1:**
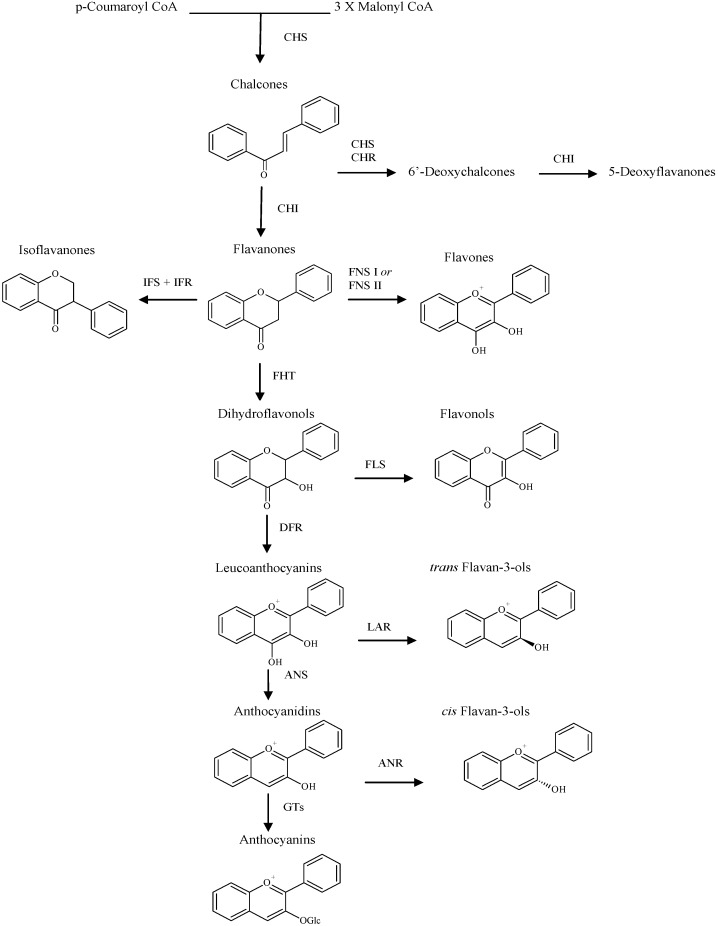
A schematic presentation of the flavonoid biosynthetic pathway showing the enzymatic steps leading to the major classes of end products. Enzymes are indicated with standard abbreviations.

Abbreviations: **ANR**, anthocyanidin reductase; **ANS**, anthocyanidin synthase (also known as leucoanthocyanidin dioxygenase); **CHI**, chalcone isomerase; **CHR**, chalcone reductase; **CHS**, chalcone synthase; **DFR**, dihydroflavonol 4-reductase; **FNSI** and **FNSII**, flavone synthase I and II; **IFR**, isoflavone reductase; **IFS**, isoflavone synthase; **LAR**, leucoanthocyanidin reductase; **GTs**, glucosyl transferases [21].

All flavonoids derive their 15-carbon skeletons (C6–C3–C6) from two basic metabolites, malonyl-CoA and *p*-coumaroyl-CoA. Their crucial biosynthetic reaction is the condensation of three molecules of malonyl-CoA with one molecule of *p*-coumaroyl-CoA to give a chalcone intermediate [21]. Chalcones act as the precursors for the vast range of flavonoid derivatives found throughout the plant kingdom. Most contain a six-membered heterocyclic ring, formed by Michael-type nucleophilic attack of a phenol group on to the unsaturated ketone giving a flavanone [22]. The first committed step of the flavonoid pathway is catalyzed by chalcone synthase (CHS; see Scheme 1). Chalcones can then be converted into aurones, a subclass of flavonoids found in certain plant species. Beyond CHS, the next step shared by most of the flavonoid biosynthesis pathways is catalyzed by chalcone isomerase (CHI), which catalyzes a stereospecific ring closure isomerization step to form the 2*S*-flavanones. The flavanones may represent the most important branching point in flavonoid metabolism, because isomerization of these compounds yields the others class of flavonoids [23]. However, the chemical synthesis is carried out mostly by cyclization and condensation of hydroxyacetophenone.

Taking into account the chemical nature of the molecule, and the positions of substituents on rings A, B, and C, the flavonoids are divided into 14 different groups [24]. Seven of these groups – the flavones, flavonols, flavanones, isoflavones, flavanols (catechins), flavanolols, and anthocyanidines – are particularly well known [24,25,26,27]. 

Flavonoids belong to the recently popular phytochemicals, chemicals derived from plant material with potentially beneficial effects on human health. The therapeutic effects of many traditional medicines may be related in many cases to the presence of these polyphenols [28]. For example, a wide variety of pharmacological activities have been reported for these substances, including antiviral [29], antiallergic [30], antiplatelet [31], antiestrogenic, anticancerogenic, anti-inflammatory, antiproliferative, antiangiogenic, and antioxidant properties, and their ingestion typically produces no or very little toxicity [24]. Flavonoids were also reported to act in the gastrointestinal tract, having antispasmodic [32], anti-secretory, antidiarrhoeal [33] and anti­ulcer properties [34]. Considering the important role of flavonoids in the prevention or reduction of gastric lesions induced by different ulcerogenic agents, this aim of this study was to review the literature on flavonoids with gastroprotective activity. The search was carried out on Pubmed, Schifinder School, Sciency Direct and NAPRALERT (Acronym for Natural Products ALERT) the data bank of The University of Illinois in Chicago, updated to December 2007, using “anti-ulcer flavonoids” as the search term. The references found in the search were later consulted for details on the models or mechanism based bioassays used for testing flavonoids against peptic ulcers.

## Flavonoids studied in models that investigate anti-ulcer activity

In this literature review, it was possible to identify ninety-five flavonoids, whose gastroprotective activities cover a full range from inactive through weak activity to active and even strong activity. Of the flavonoids found in this study, forty-two were reportedly inactive; however, this inactivity could vary widely according to the experimental model, animal, route of administration and the dose. For example, flavonols like kaempferol, robinin and dactailin showed no gastroprotective effect in experimental models of reserpine [35,36] and restraint stress-induced ulcers in mouse [35], but kaempferol at doses of 50 and 100 mg/kg showed gastroprotective activity, and when the dose was increased to 250 mg/kg, it showed no activity [37]. Similar results were found for nobeletin, a flavone, where doses of 8 and 25 mg/kg protect the gastric mucosa of the rats from injuries induced by ethanol and HCl/ethanol, respectively, but it was only weakly active at a dose of 50 mg/kg in model of aspirin-induced ulcers [38]. Although many of the pharmacological and biochemical actions of flavonoids are attributed to their activities as antioxidants [39], this observed inactivity in high doses may be related to the capacity of flavonoids to act as pro-oxidants. Thus, flavonoids like quercetin, myricetin and kaempferol induce a concentration-dependent decrease of both the nuclear glutathione (GSH) content and glutathione S-transferase (GST) activity in a model system of isolated rat liver nuclei, which could lead to oxidative DNA damage [40], which in turn may be responsible for their mutagenicity and carcinogenicity; this effect may be explained by the pro-oxidant effects of this compounds [40, 41]. Nevertheless, the structural features that might determine the pro-oxidant activity of these compounds are not well established.

Chalcones belong to flavonoid class with the largest number of compounds with gastroprotective activity. In this review were found thirty-eight, among which we can mention sophoradin, an isoprenyl chalcone, which is present in a Chinese crude drug (the root of *Sophora subprostrata*) and protects the gastric mucosa from lesions induced by pylorus-ligation and water-immersion stress [42,43]. Thirty sophoradin analogs have shown anti-ulcer effects in the same ulcer induction models. Several chalcones, all having more than one isoprenyloxyl group, exhibited high inhibitory ratios. In particular, 2’,4’-dihydroxy-3’-(3-methyl-2-butenyl)-4-(3-methyl-2-butenyloxy) chalcone, 2’-hydroxy-4,4’-bis(3-methyl-2-butenyloxy) chalcone and 2’-carboxymethoxy-4,4’-bis(3-methyl-2-butenyloxy) chalcone (sofalcone), showed strong activity at a dose of 100 mg/kg, with a high percentage of inhibition of lesions (70-100%), when compared to other chalcones at the same dose and were as potent as sophoradin [42]. Sofalcone is one of these analogs that in addition to its gastroprotective effects also accelerates ulcer healing [44]. The mechanisms of action involved in gastric protection are increased gastric blood flow, stimulated synthesis of mucosubstances of the gastric mucosa [45] and increasing effects on gastric tissue PGs contents [46]. Besides its cytoprotective effects, sofalcone has a direct bactericidal effect on *H. pylori*, with a minimum inhibitory concentration of 55-222 µmol/L, anti-urease activity and it reduces the adhesion of this organism to gastric epithelial cells [47,48]. When outpatients with peptic ulcers and *H. pylori* infections were medicated for 7 d with sofalcone (100 mg thrice daily) plus the triple therapy with rabeprazole (10 mg twice daily), clarithromycin (200 mg twice daily) and amoxicillin (750 mg twice daily), sofalcone significantly increased the cure rate of *H. pylori* infections [49]. Therefore flavonoids can be utilized as alternative or additive agents to the current therapy in treatment of peptic ulcer induced by *H. pylori* infection.

Another flavonoid that appears to exert anti-ulcer activity is monomeric leucocyanidin, a natural flavonoid and the major component present in unripe plantain banana (*Musa sapientum L*. v*ar*. paradisiaca). It and its synthetic analogues hydroxyethylated leucocyanidin and tetrallylleucocyanidin showed protective effects against aspirin-induced gastric erosions in a prophylactic animal model, as shown by the absence of mucosal damage and a significant reduction in the ulcer index, when added to the diet at 5 mg and 15 mg per day [50,51]. The authors concluded that these compounds may be responsible for the displayed anti-ulcer properties and they suggested that the mechanism by which the active agent present in plantain banana and its synthetic analogues protects the mucosa is mediated, at least in part, by an increase in mucus thickness [51].

Another polyphenolic compound with relevant activities is garcinol, a polyisoprenylated benzophenone derivative from *Garcinia indica*, which shows potent free radical scavenging activity in three kinds of free radical generating systems. In the hypoxanthine/xanthine oxidase system, emulsified garcinol suppressed superoxide anion to almost the same extent as dl-α-tocopherol by weight and also suppressed hydroxyl radical more strongly than dl-α-tocopherol in the Fenton reaction system. In the H_2_O_2_/NaOH/DMSO system, this compound suppressed superoxide anion, hydroxyl radical, and methyl radical. Orally administered garcinol prevented acute ulceration in rats induced by indomethacin (40-200 mg/kg) and water immersion stress (200 mg/kg) caused by radical formation. These results suggested that garcinol might have potential as a free radical scavenger and clinical applications as an anti-ulcer drug. Although the mechanism of its anti-ulcer activity is not yet understood, garcinol may scavenge reactive oxygen species on the surface of gastric mucosa, thus protecting cells from injury [52].

A flavonoid that has been studied in some detail is rutin (quercetin-3-rhamnosylglucoside), a natural flavone derivative. It has been reported to prevent gastric mucosal ulceration in animal models including reserpine [35], acidified ethanol [37] and absolute and 50% ethanol [34,37]. The cytoprotective effect of this flavonoid does not appear to be mediated by endogenous prostaglandins [53], but its protective effects may be mediated by endogenous platelet-activating factor (PAF), since it inhibited dose-dependently the mucosal content of PAF [37]. Another possible mechanism involves the antioxidant properties of rutin, which at a dose of 200 mg/kg has a protective effect against lesions induced by 50 % ethanol, probably by reducing the levels of lipoperoxides and increasing the activity of the antioxidant enzyme glutathione peroxidase (GSH-Px). However, no significant modifications were observed in the gastric non-protein sulfhydryl (SH) content or in the ethanol-induced leukocyte infiltrate [34]. 

One of the most studied flavonoids is quercetin (3,3’,4’,5,7-pentahydroxyflavone). It protects the gastrointestinal mucosa from acute lesions induced by various experiemental models and against different necrotic agents, including restraint stress [37,54,55] pylorus-ligation [56], reserpine [35,36,55,57], aspirin [54], indomethacin [58], acid-ethanol [37] and ethanol-induced gastric ulcers [54,59,60]. Its gastroprotective action mechanism involves endogenous PAF [37], an increase in mucus production [58], antihistaminic properties, which decrease histamine levels and reduction of the number of ethanol-induced mast cells. It also inhibits *H. pylori* growth, the formation of acid by parietal cells in response to stimulation by histamine and dibutyryl cyclic AMP, as well as the gastric H^+^/K^+^ proton pump (data not shown in Table 1) [61]. The main mechanism of action for the gastroprotective effects of this flavonol are its antioxidant properties, since oral pretreatment with quercetin (200 mg/kg) had protective effects in that it significantly reduced the severity of ethanol-induced ulcers by inhibition of lipid peroxidation, enhancement in the levels of mucosal non-protein SH compounds (important antioxidant agents) [59,60] in GSH-Px [59] and superoxide dismutase activities, as well as reduction of protein carbonyl compounds [60]. At a dose of 100 mg/kg twice daily for 5 days it also decreases lipid peroxidation and plasmatic corticosterone in a restraint stress model. This flavonoid, in addition to protecting the gastric mucosa in acute models of ulcer induction, when administered chronically both quercetin and naringenin also promote healing of gastric ulcers induced by acetic acid, a chronic model of ulcer [62]. The antioxidant mechanism of action of flavonoids, especially garcinol, rutin and quercetin, is due mainly the presence in their structures of an o-dihydroxy in the B ring (catechol), and additionally a 2,3 double bond in conjugation with a 4-oxo function, as well as the presence of hydroxyl groups in positions 3, 5 and 7 [24,63,64]

Finally, nowadays it is known that NSAIDs, such as piroxicam or aspirin have several adverse effects on the gastrointestinal tract and increase the risk of myocardial infarction. However, several flavonoids have demonstrated anti-inflammatory properties, without showing any ulcerogenic action as a side effect, and thus showing a great advantage in the treatment of peptic ulcers.

**Table 1:** Flavonoids with gastroprotective activity.

Substance	Experimental assay/Administration route	Animal tested	Dose	Activity
**Chalcones**				
Butein 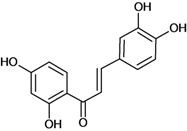	HCl/ethanol-induced ulcers/intragastric	Rat	10 mg/kg	Active [65]
	NaOH-induced ulcers/intragastric	Rat	50.0 mg/kg	Inactive [65]
2',3,4,4',6'-pentahydroxychalcone 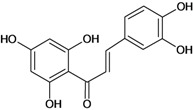	HCl/ethanol-induced ulcers/intragastric	Rat	10.0 mg/kg	Active [65]
	NaOH-induced ulcers/intragastric	Rat	10.0 mg/kg	Active [65]
2',3,4-trihydroxychalcone 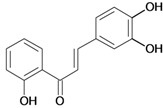	HCl/ethanol-induced ulcers/intragastric	Rat	10.0 mg/kg	Active [65]
	NaOH-induced ulcers/intragastric	Rat	10.0 mg/kg	Active [65]
2',4',6'-trihydroxychalcone 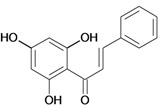	HCl/ethanol-induced ulcers/intragastric	Rat	10.0 mg/kg	Active [65]
	NaOH-induced ulcers/intragastric	Rat	10.0 mg/kg	Active [65]
2',4'-dihydroxy-3',5'-diprenyl-4-O-prenyl- chalcone 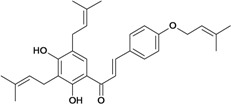	Stress-induced ulcers (water-immersion)/i.p.	Rat	100.0 mg/kg	Active [42]
2',4'-dihydroxy-3'-methoxychalcone 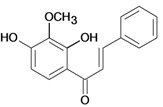	Ethanol-induced ulcers/intragastric	Mouse	*	Weak Active [66]
	Ethanol-induced ulcers/intragastric	Rat	100.0 mg/kg	Active [67]
	Ethanol-induced ulcers/intragastric	Rat	*	Active [66]
2',4'-dihydroxy-5'-prenyl-4-O-prenyl- chalcone 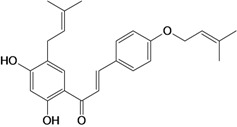	Pylorus ligation-induced ulcers/i.p.	Rat	100.0 mg/kg	Active [42]
	Stress-induced ulcers (water-immersion)/i.p.	Rat	100.0 mg/kg	Active [42]
2',4'-dihydroxychalcone 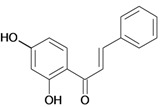	Stress-induced ulcers (water-immersion)/intragastric	Rat	10.0 mg/kg	Active [65]
	Acetic acid-induced ulcers/intragastric	Rat	10.0 mg/kg	Active [65]
	HCl/ethanol-induced ulcers/intragastric	Rat	10.0 mg/kg	Active [65]
	NaOH-induced ulcers/intragastric			
	Ethanol-induced ulcers/intragastric	Rat	10.0 mg/kg	Active [65]
	Ethanol-induced ulcers/intragastric	Mouse	*	Active [66]
	Ethanol-induced ulcers/intragastric	Rat	100 mg/kg	Active [67]
		Rat	*	Active [66]
2',4,4',6'-tetrahydroxychalcone 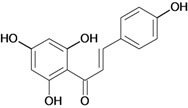	HCl/ethanol-induced ulcers/intragastric	Rat	10.0 mg/kg	Inactive [65]
	NaOH-induced ulcers/intragastric	Rat	10.0 mg/kg	Active [65]
2',4,4'-trihydroxy-3,3',5'-tris-(3-methyl-but-2-enyl) chalcone 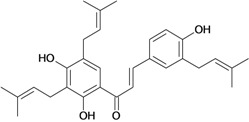	*/*	Rat	*	Active [68]
2',4,4'-trihydroxy-3,3',5,5'-tetrakis-(3-methyl-but-2-enyl)-4,4'-bis-(O-3-methyl-but-2-enyl) chalcone 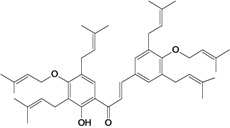	*/*	Rat	*	Active [69]
2',4,4'-trihydroxy-3,3',5,5'-tetrakis-3-methyl-but-2-enyl) chalcone 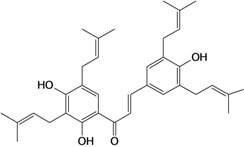	*/*	Rat	*	Active [68]
2',4,4'-trihydroxy-3,3',5-tris-(3-methyl-but-2-enyl)-4-4'-di-O-allyl chalcone 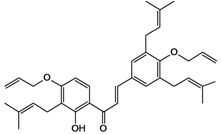	*/*	Rat	*	Active [69]
2',4,4'-trihydroxy-3,3'-bis-(3-methylbut-2-enyl) chalcone 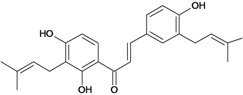	*/*	Rat	*	Active [68]
2',4,4'-trihydroxy-3,3'-diprenylchalcone 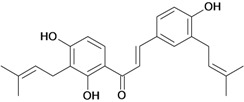	Stress-induced ulcers (water-immersion)/i.p.	Rat	100.0 mg/kg	Active [42]
2',4,4'-trihydroxy-3,5,5'-tris-(3-methyl-but-2-enyl)-4'-O-(3-methylbut-2-enyl) chalcone 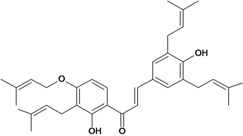	*/*	Rat	*	Active [69]
2',4,4'-trihydroxychalcone 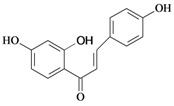	HCl/ethanol-induced ulcers/intragastric	Rat	10.0 mg/kg	Active [65]
	NaOH-induced ulcers/intragastric	Rat	10.0 mg/kg	Active [65]
2',4-dihydroxy-3-prenyl-4'-O-prenyl- chalcone 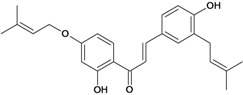	Stress-induced ulcers (water-immersion)/i.p.	Rat	100.0 mg/kg	Active [42]
2',4-dihydroxy-4'-methoxy-3-5-bis-(3-methyl-but-2-enyl) chalcone 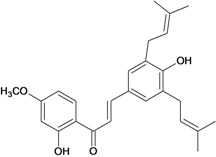	*/*	Rat	*	Active [42]
2'-carbomethoxy-4,4'-bis-(3-methyl-2-butenyl-oxy) chalcone (sofalcone) 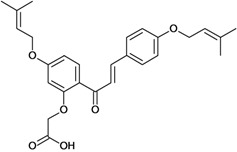	Histamine-induced ulcers/i.p.	Rat	100.0 mg/kg	Active [45]
	Acetic acid-induced ulcers/gastric intubation	Rat	20-50 mg/kg	Active [45]
	Histamine-induced ulcers/gastric intubation	Guinea pig	100.0 mg/kg	Active [45]
	Pylorus ligation-induced ulcers/i.p.	Rat	50.0 mg/kg	Active [45]
	Stress-induced ulcers (water-immersion)/i.p.	Rat	50.0 mg/kg	Active [45]
	Phenylbutazone-induced ulcers/gastric ntubation	Rat	300.0 mg/kg	Active [45]
	Acetic acid-induced ulcers/gastric intubation	Rat	50.0 mg/kg	Active [44]
	HCl induced gastric lesions/i.p.	Rat	100.0 mg/kg	Active [46]
	HCl induced gastric lesions/gastric intubation	Rat	100.0 mg/kg	Active [46]
	Pretreatment with indomethacin vs HCl induced gastric lesions/gastric intubation	Rat	300.0 mg/kg	Active [46]
	Pretreatment with indomethacin vs HCl induced gastric lesions/i.p.	Rat	100.0 mg/kg	Active [46]
	*H. pylor*i induced ulcer/p.o.	Human adult	100.0 mg/kg	Active [49]
2'-hydroxy-4,4'-di-O-prenylchalcone 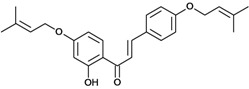	Pylorus ligation-induced ulcers/i.p.	Rat	100.0 mg/kg	Strong activity [42]
	Stress-induced ulcers (water-immersion)/i.p.	Rat	100.0 mg/kg	Strong activity [42]
2,4'-di-O-prenylchalcone 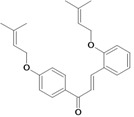	Pylorus ligation-induced ulcers/i.p.	Rat	100.0 mg/kg	Active [42]
	Stress-induced ulcers (water-immersion)/i.p.	Rat	100.0 mg/kg	Weak activity [42]
2,4,4'-trihydroxy-3,3',5'-tris-(3-methyl-but-2-enyl)-4-O-allyl-4-O-propargyl-chalcone 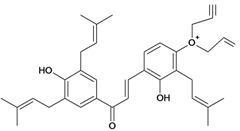	*/*	Rat	*	Active [69]
3',5'-dihydroxy-4'-prenyl-5-O-prenyl- chalcone 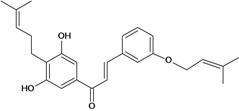	Pylorus ligation-induced ulcers/i.p.	Rat	100.0 mg/kg	Strong activity [42]
	Stress-induced ulcers (water-immersion)/i.p.	Rat	100.0 mg/kg	Strong activity [42]
3,3',4-trihydroxychalcone 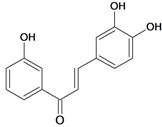	HCl/ethanol-induced ulcers/intragastric	Rat	10.0 mg/kg	Active [65]
	NaOH-induced ulcers/intragastric	Rat	10.0 mg/kg	Active [65]
3,4,4'-trihydroxychalcone 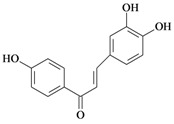	HCl/ethanol-induced ulcers/intragastric	Rat	10.0 mg/kg	Inactive [65]
	NaOH-induced ulcers/intragastric	Rat	10.0 mg/kg	Active [65]
4'-hydroxy-3'-prenyl-4-O-prenylchalcone 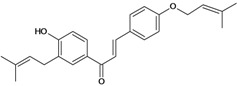	Pylorus ligation-induced ulcers/i.p.	Rat	100.0 mg/kg	Active [42]
	Stress-induced ulcers (water-immersion)/i.p.	Rat	100.0 mg/kg	Strong activity [42]
4,4'-di-O-geranyl chalcone 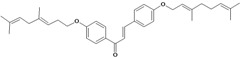	Pylorus ligation-induced ulcers/i.p.	Rat	100.0 mg/kg	Weak activity [42]
	Stress-induced ulcers (water-immersion)/i.p.	Rat	100.0 mg/kg	Weak activity [42]
4,4'-di-O-prenylchalcone 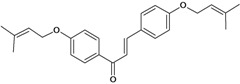	Pylorus ligation-induced ulcers/i.p.	Rat	100.0 mg/kg	Active [42]
	Stress-induced ulcers (water-immersion)/i.p.	Rat	100.0 mg/kg	Strong activity [42]
4,4'-dihydroxy-3,3'-diprenylchalcone 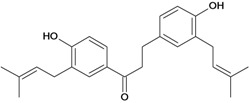	Stress-induced ulcers (water-immersion)/i.p.	Rat	100.0 mg/kg	Active [42]
4,4'-dimethoxy-3,3'-diprenylchalcone 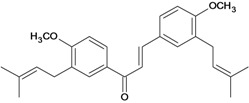	Pylorus ligation-induced ulcers/i.p.	Rat	100.0 mg/kg	Weak activity [42]
	Stress-induced ulcers (water-immersion)/i.p.	Rat	100.0 mg/kg	Active [42]
4-hydroxy-3-prenyl-4'-O-prenylchalcone 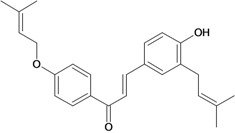	Pylorus ligation-induced ulcers/i.p.	Rat	100.0 mg/kg	Active [42]
	Stress-induced ulcers (water-immersion)/i.p.	Rat	100.0 mg/kg	Weak activity [42]
2',4-bis-(carbomethoxy)-4'-(3-carboxy-2-butenyl-oxy) dihydrochalcone 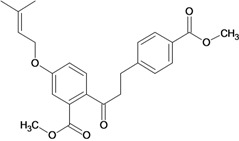	Pylorus ligation-induced ulcers/i.p.	Rat	100.0 mg/kg	Weak activity [70]
	Stress-induced ulcers (water-immersion)/i.p.	Rat	100.0 mg/kg	Weak activity [70]
	Histamine-induced ulcers/i.p.	Rat	100.0 mg/kg	Weak activity [70]
2',4-bis-(carboxymethoxy)-4'-(3-methyl-2-butenyl-oxy) dihydrochalcone 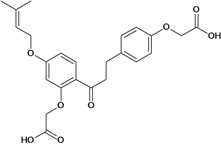	Pylorus ligation-induced ulcers/i.p.	Rat	100.0 mg/kg	Active [70]
	Stress-induced ulcers (water-immersion)/i.p.	Rat	100.0 mg/kg	Weak activity [70]
	Histamine-induced ulcers/i.p.	Rat	100.0 mg/kg	Active [70]
2'-carboxymethoxy-4-4'-bis-(3-methyl-2-butenyl-oxy) dihydro-chalcone 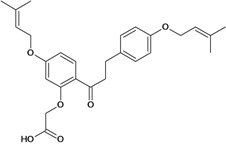	Pylorus ligation-induced ulcers/i.p.	Rat	100.0 mg/kg	Active [70]
	Stress-induced ulcers (water-immersion)/i.p.	Rat	100.0 mg/kg	Active [70]
	Histamine-induced ulcers/i.p.	Rat	100.0 mg/kg	Active [70]
Garcinol 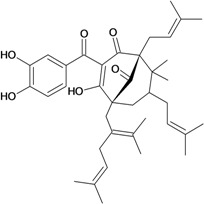	Stress-induced (restraint) ulcers/intragastric	Rat	200.0 mg/kg	Active [52]
	Indomethacin-induced ulcers/intragastric	Rat	200.0 mg/kg	Active [52]
Sophoradin 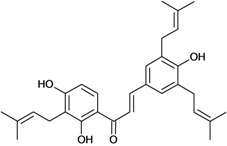	Pylorus ligation-induced ulcers/p.o.	Rat	*	Active [43]
	Stress-induced ulcers/p.o.	Rat	*	Active [43]
	Pylorus ligation-induced ulcers/p.o.	Rat	100.0 mg/kg	Strong activity [42]
	Stress-induced ulcers/p.o.	Rat	100.0 mg/kg	Strong activity [42]
Xanthoangelol E 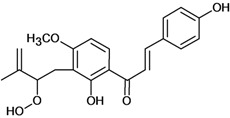	Stress-induced (restraint) ulcers/intragastric	Rat	100.0 mg/kg	Active [71]
**Flavanones**				
3',4',5,7-tetrahydroxy-3-methoxy- flavanone 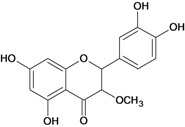	stress-induced (restraint) ulcers/*	Rat	*	Active [72]
2',4',7-trihydroxy-5-methoxy-8-(5-hydroxy-5-methyl-2-iso-propenyl-hexyl) flavanone 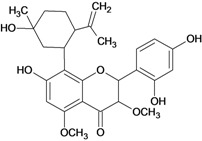	*/p.o.	Human adult	*	Active [73]
Hesperidin 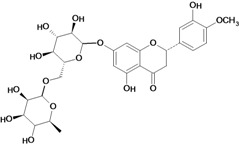	Cold stress-induced ulcers/intragastric	Rat	100.0 mg/kg	Active [74]
	Ethanol-induced ulcers/intragastric	Rat	100.0 mg/kg	Inactive [74]
Naringenin 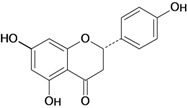	Acetic acid-induced ulcers/intragastric	Rat	100.0 mg/kg	Active [62]
	Stress-induced ulcers (water-immersion)/intragastric	Rat	100.0 mg/kg	Weak active [56]
	Pylorus ligation-induced ulcers/intragastric	Rat	100.0 mg/kg	Active [56]
	Pylorus ligation-induced ulcers /intragastric	Rat	100.0 mg/kg	Active [56]
	Pylorus ligation-induced ulcers/gastric intubation	Rat	ED50 132 mg/kg	Active [75]
	Stress-induced (restraint) ulcers/gastric intubation	Rat	ED50 42.0 mg/kg	Active [75]
	Aspirin-induced ulcers/gastric intubation	Rat	*	Active [75]
	Phenylbutazone-induced ulcers/gastric intubation	Rat	*	Active [75]
	Reserpine-induced ulcers/gastric intubation	Rat	*	Active [75]
Naringin 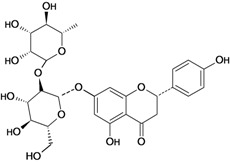	Aspirin-induced ulcers/intragastric	Rat	200.0 mg/kg	Active [76]
	Acid-ethanol-induced ulcers/i.p.	Rat	100.0 mg/kg	Inactive [37]
	Acid-ethanol-induced ulcers/i.p.	Rat	200.0 mg/kg	Active [37]
	Acid-ethanol-induced ulcers/i.p.	Rat	400.0 mg/kg	Active [37]
	Ethanol-induced gastric injury/intragastric	Rat	400.0 mg/kg	Active [37]
Sigmoidin A 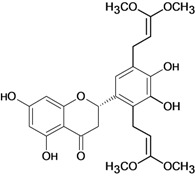	Stress-induced ulcers (water-immersion)/gastric intubation	Rat	50.0 mg/kg	Active [78]
	Stress-induced (restraint) ulcers/gastric intubation	Rat	50.0 mg/kg	Active [78]
Sigmoidin B 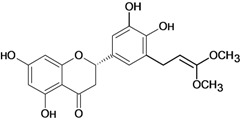	Stress-induced ulcers (water-immersion)/gastric intubation	Rat	50.0 mg/kg	Active [78]
	Stress-induced (restraint) ulcers/gastric intubation	Rat	50.0 mg/kg	Active [78]
Sophoranone 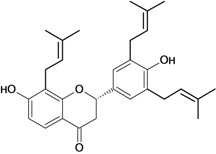	Pylorus ligation-induced ulcers/p.o.	Rat	*	Active [43]
	Stress-induced ulcers/p.o.	Rat	*	Active [43]
**Flavane and Flavanols**				
(+) catechin 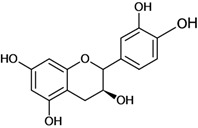	HCl/ethanol-induced stomach ulcers/intragastric	Rat	*	Inactive [79]
	*/pathway oral (p.o.)	Rat	100.0 mg/kg	Active [80]
	Reserpine-induced ulcers/gastric intubation	Mouse	49.7 mg/kg	Equivocal [57]
	Reserpine-induced ulcers/gastric intubation	Mouse	72.5 mg/kg	Inactive [57]
	Stress-induced ulcers (water-immersion)/gastric intubation	Mouse	500.0 mg/kg	Weak Active [81]
(dl) catechin 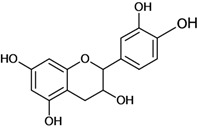	Stress-induced (restraint) ulcers/subcutaneous (s.c.)	Mouse	300.0 mg/kg	Active [82]
	Stress-induced (restraint) ulcers/intragastric	Mouse	300.0 mg/kg	Active [82]
	Stress-induced ulcers (water-immersion)/s.c.	Mouse	300.0 μmol/kg	Active [82]
	Stress-induced ulcers (water-immersion)/intragastric	Mouse	300.0 mg/kg	Active [82]
3-O-methyl: (+) catechin 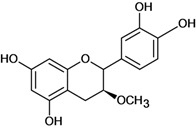	Pylorus ligation-induced ulcers/s.c.	Rat	ED50 60.0 mg/kg	Active [83]
	Stress-induced (restraint) ulcers/s.c.	Rat	ED50 13.2 mg/kg	Active [83]
	*/s.c.	Rat	*	Active [83]
	Phenylbutazone-induced ulcers/s.c.	Rat	*	Active [83]
	Reserpine-induced ulcers/s.c.	Rat	*	Active [83]
	*/*	*	*	Active [84]
(-) Epicatechin 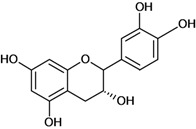	Stress-induced ulcers (water-immersion)/gastric intubation	Mouse	500.0 mg/kg	Weak Active [81]
(+) Cyanidan-3-beta- ol 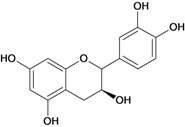	Pylorus ligation-induced ulcers/s.c.	Rat	ED_50_ 62 mg/kg	Active [85]
	Restraint-induced ulcers/s.c.	Rat	ED_50_ 18 mg/kg	Active [85]
	Aspirin-induced ulcers/gastric intubation	Rat	100.0 mg/kg	Active [85]
	Phenylbutazone-induced ulcers/gastric intubation	Rat	100.0 mg/kg	Active [85]
	Ibuprofen-induced ulcers/gastric intubation	Rat	100.0 mg/kg	Active [85]
	Reserpine-induced ulcers/gastric intubation	Rat	100.0 mg/kg	Active [85]
Leucocyanidin 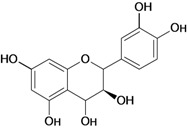	Aspirin-induced ulcers/*	Rat	5.0 mg/day	Active [50]
	*/intragastric	Rat	*	Active [51]
**Flavanolols**				
Taxifolin 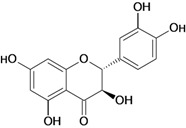	Ethanol induced gastric ulcers/intragastric	Rat	50.0 mg/kg	Active [86]
Taxifolin,(dl) 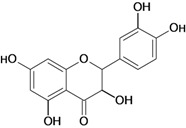	HCl /ethanol-induced stomach ulcers/intragastric	Rat	* mg/kg	Inactive [79]
**Anthocyanidines**				
Benzopyrylium chloride,1: 3,5,7- trihydroxy-2-(3-4-dihydroxyphenyl) 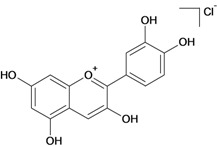	Pylorus ligation-induced ulcers/intragastric	Rat	12.5 mg/kg	Active [87]
	Stress-induced (restraint) ulcers/intragastric	Rat	100.0 mg/kg	Active [87]
	Phenylbutazone-induced ulcers/intragastric	Rat	22.0 mg/kg	Active [87]
	Indomethacin-induced ulcers/intragastric	Rat	100.0 mg/kg	Active [87]
	Reserpine-induced ulcers/intragastric	Rat	100.0 mg/kg	Active [87]
	Ethanol induced lesion/intragastric	Rat	200.0 mg/kg	Active [87]
	Histamine-induced ulcers/intragastric	Rat	24.0 mg/kg	Active [87]
	Cysteamine-induced ulcers/intragastric	Rat	200.0 mg/kg	Active [87]
	Cysteamine-induced ulcers/intraperitoneal (i.p.)	Rat	50.0 mg/kg	Active [87]
	Acetic acid-induced ulcers/intragastric	Rat	50.0 mg/kg	Active [87]
**Flavones**				
Acacetin 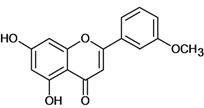	Reserpine-induced ulcers/gastric intubation	Mouse	0.05 mL/g	Inactive [36]
Apigenin 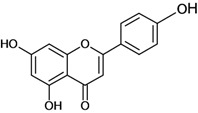	Reserpine-induced ulcers/gastric intubation	Mouse	0.05 mL/g	Inactive [36]
Cynaroside 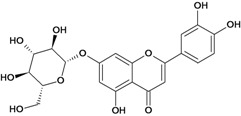	*/intragastric	Rat	*	* [88]
Dactylin 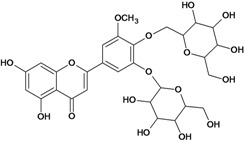	Reserpine-induced ulcers/gastric intubation	Mouse	*	Inactive [35]
	Stress-induced (restraint) ulcers/gastric intubation	Mouse	*	Inactive [35]
Eupatilin 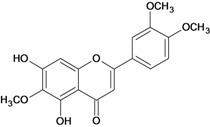	*/ Intragastric	Rat	*	Active [89]
Gnaphaloside A 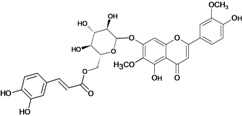	Reserpine-induced ulcers/gastric intubation	Mouse	0.05 mL/g	Active [36]
Gossypin 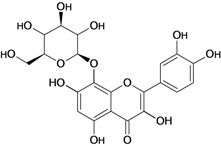	*/oral	Rat	100.0 mg/kg	Active [80]
Hyperoside 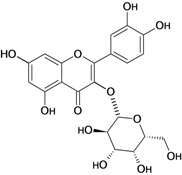	Reserpine-induced ulcers/gastric intubation	Mouse	*	Weak activity [35]
	Stress-induced (restraint) ulcers/gastric intubation	Mouse	*	Weak activity [35]
Hypolaetin-8-O-beta-d-glucoside 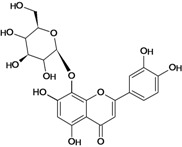	Cold stress-induced ulcers/i.p.	Rat	ED50 573mg/kg	Active [90]
	Cold stress-induced ulcers/*	*	ED50 57.3mg/kg	Active [91]
	Ethanol-induced gastric lesions/s.c.	Rat	ED50 68.0mg/kg	Active [92]
Kaempferol rhamnoside 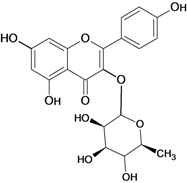	Reserpine-induced ulcers/gastric intubation	Mouse	0.05 mL/g	Active [36]
Linarin 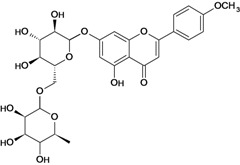	Reserpine-induced ulcers/gastric intubation	Mouse	0.05 mL/g	Inactive [36]
Luteolin 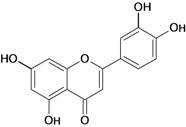	*/intragastric	Rat	*	Active [88]
	Reserpine-induced ulcers/gastric intubation	Mouse	47.4 mg/kg	Active [57]
	Reserpine-induced ulcers/gastric intubation	Mouse	474 mg/kg	Active [57]
Myricetin rhamnoside 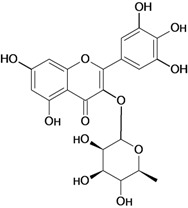	Reserpine-induced ulcers/gastric intubation	Mouse	0.05 mL/g	Active [36]
Nobiletin 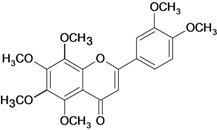	Ethanol-induced gastric ulcer/intragastric	Rat	ED_50_ 8.0 mg/kg	Active [38]
	Ethanol-induced ulcers/intragastric	Rat	ED_50 _8.0 mg/kg	Active [93]
	Aspirin-induced ulcers/intragastric	Rat	50.0 mg/kg	Weak active [93]
	HCl/ethanol-induced gastric ulcers/intragastric	Rat	25.0 mg/kg	Active [97]
Pectolinarigenin 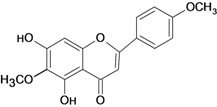	Reserpine-induced ulcers/gastric intubation	Mouse	0.05 mL/g	Inactive [36]
Pectolinarin 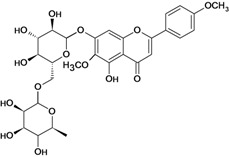	Reserpine-induced ulcers/gastric intubation	Mouse	0.05 mL/g	Inactive [36]
Acetyl pectolinarin 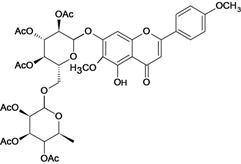	Reserpine-iduced ulcers/gastric itubation	Mouse	0.05 mL/g	Inactive [36]
Quercetin rhamnoside 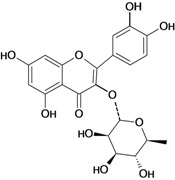	Reserpine-induced ulcers/gastric intubation	Mouse	0.05 mL/g	Active [36]
Quercitrin 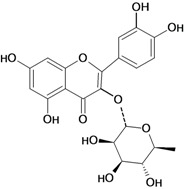	Reserpine-induced ulcers/gastric intubation	Mouse	50.0 mg/g	Active [57]
Robinin 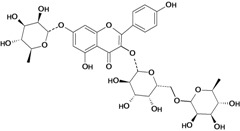	Reserpine-induced ulcers/gastric intubation	Mouse	*	Inactive [36]
	Stress-induced (restraint) ulcers/gastric intubation	Mouse	*	Inactive [36]
Rutin 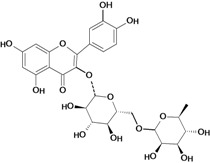	Acid-ethanol-induced ulcers/i.p.	Mouse	12.5 mg/kg	Inactive [37]
	Acid-ethanol-induced ulcers/i.p.	Rat	25.0 mg/kg	Active [37]
	Acid-ethanol-induced ulcers/i.p.	Rat	50.0 mg/kg	Active [37]
	Pretreatment with indomethalin vs ethanol induced-ulcers/intragastric	Rat	25.0 mg/kg	Weak activity [53]
	Ethanol-induced ulcers/intragastric	Rat	50.0 mg/kg	Active [53]
	Ethanol-induced ulcers/ intragastric	Rat	200.0 mg/kg	Active [34]
	*/intragastric	Mouse	7.0 mg/kg	Active [95]
	*/intragastric	Mouse	*	Active [96]
	Reserpine-induced ulcers/gastric intubation	Mouse	*	Weak activity [35]
	Stress-induced (restraint) ulcers/gastric intubation	Mouse	*	Weak activity [35]
Salvigenin 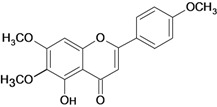	Pylorus ligation-induced ulcers/i.p.	Rat	100.0 mg/kg	Inactive [97]
Scoparin 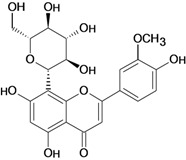	Reserpine-induced ulcers/gastric intubation	Mouse	0.05 mL/g	Inactive [36]
Ternatin 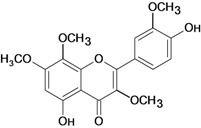	Cold stress-induced ulcers/i.p.	Rat	25.0 mg/kg	Inactive [98]
	Ethanol-induced ulcers/i.p.	Rat	25.0 mg/kg	Inactive [98]
	Indomethacin-induced ulcers/i.p.	Rat	25.0 mg/kg	Inactive [98]
Vexibinol 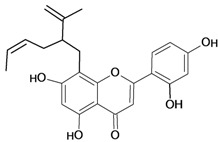	HCl-ethanol induced ulcers/intragastric	Rat	10.0 mg/kg	Active [99]
	Stress-induced ulcers (water-immersion)/intragastric	Rat	10.0 mg/kg	Active [99]
	Pylorus ligation-induced ulcers/intragastric	Rat	100.0 mg/kg	Active [99]
	Indomethacin-induced ulcers/intragastric	Rat	100.0 mg/kg	Active [99]
	Histamine-induced ulcers/intragastric	Rat	100.0 mg/kg	Inactive [99]
	5-Ht-induced ulcers/intragastric	Rat	300.0 mg/kg	Inactive [99]
	Phenylbutazone induced ulcers/intragastric	Rat	300.0 mg/kg	Active [99]
**Isoflavones**				
Genistin 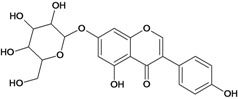	*/intragastric	Rat	*	Active [88]
**Flavonols**				
Kaempferol 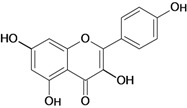	Acid-ethanol-induced ulcers/i.p.	Rat	250.0 mg/kg	Inactive [37]
	Acid-ethanol-induced ulcers/i.p.	Rat	50.0 mg/kg	Active [37]
	Acid-ethanol-induced ulcers/i.p.	Rat	100.0 mg/kg	Active [37]
	Ethanol-induced ulcers/i.p.	Rat	100.0 mg/kg	Active [100]
	Cold stress-induced ulcers/i.p.	Rat	200.0 mg/kg	Active [100]
	Reserpine-induced ulcers/gastric intubation	Mouse	0.05 mL/g	Inactive [36]
	Pylorus ligation-induced ulcers/i.p.	Rat	200.0 mg/kg	Active [101]
	Stress-induced (restraint) ulcers/i.p.	Rat	200.0 mg/kg	Active [101]
	Reserpine-induced ulcers/gastric intubation	Mouse	*	Inactive [35]
	Stress-induced (restraint) ulcers/gastric	Mouse	*	Inactive [35]
Myricetin 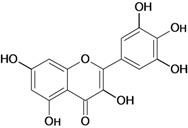	Reserpine-induced ulcers/gastric intubation	Mouse	0.05 mL/g	Inactive [36]
	Reserpine-induced ulcers/gastric intubation	Mouse	0.05 mL/g	Active [36]
	Reserpine-induced ulcers/gastric intubation	Mouse	*	Active [55]
	Stress-induced (restraint) ulcers/gastric intubation	Mouse	*	Active [55]
	Reserpine-induced ulcers/gastric intubation	Mouse	*	Active [35]
	Stress-induced (restraint) ulcers/gastric intubation	Mouse	*	Active [35]
Patuletin 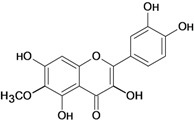	Reserpine-induced ulcers/gastric intubation	Mouse	0.05 mL/g	Inactive [36]
Patulitrin 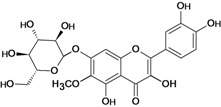	Reserpine-induced ulcers/gastric intubation	Mouse	0.05 mL/g	Weak active [36]
Phellavin 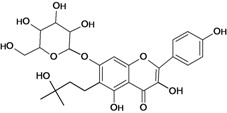	Reserpine-induced ulcers/gastric intubation	Mouse	0.05 mL/g	Inactive [36]
Quercetin 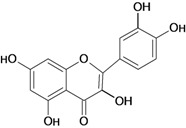	Ethanol-induced gastric lesions/intragastric	Rat	200.0 mg/kg	Active [59]
	Acetic acid-induced ulcers/intragastric	Rat	100.0 mg/kg	Active [62]
	Stress-induced ulcers (water-immersion)/intragastric	Rat	100.0 mg/kg	Active [56]
	Pylorus ligation-induced ulcers/intragastric	Rat	100.0 mg/kg	Active [56]
	Pylorus ligation-induced ulcers/intragastric	Rat	100.0 mg/kg	Active [56]
	*/intragastric	Rat	200.0 mg/kg	Active [58]
	Acid-ethanol-induced ulcers/i.p.	Rat	12.5 mg/kg	Inactive [37]
	Acid-ethanol-induced ulcers/i.p.	Rat	25.0 mg/kg	Active [37]
	Acid-ethanol-induced ulcers/i.p.	Rat	50.0 mg/kg	Active [37]
	Ethanol-induced gastric ulcers/i.p.	Rat	12.5 mg/kg	Active [104]
	*/ intragastric	Mouse	*	Active [96]
	Ethanol-induced ulcers/intragastric	Rat	100.0 mg/kg	Active [54]
	Stress-induced (restraint) ulcers/intragastric	Rat	100.0 mg/kg	Active [54]
	Ethanol-induced ulcers/intragastric	Rat	200.0 mg/kg	Active [60]
	Reserpine-induced ulcers/gastric intubation	Mouse	0.05 mL/gm	Inactive [36]
	Reserpine-induced ulcers/gastric intubation	Mouse	50.0 mg/kg	* [57]
	Reserpine-induced ulcers/gastric intubation	Mouse	*	Active [55]
	Stress-induced (restraint) ulcers/gastric	Mouse	*	Active [55]
	Reserpine-induced ulcers/gastric intubation	Mouse	*	Active [35]
	Stress-induced (restraint) ulcers/gastric intubation	Mouse	*	Active [35]
Quercetin-3'-o-beta-d-glucoside 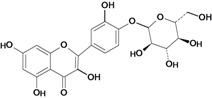	Reserpine-induced ulcers/gastric intubation	Mouse	0.05 mL/g	Inactive [36]
**Biflavonoids**				
Cinnamtannin B-1 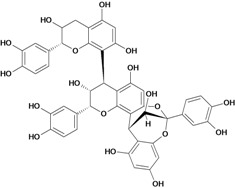	Stress-induced ulcers (water-immersion)/gastric intubation	Mouse	500.0 mg/kg	Inactive [81]
Cinnamtannin D-1 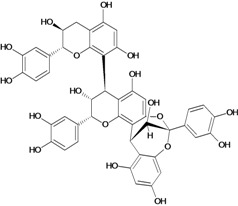	Stress-induced ulcers (water-immersion)/gastric intubation	Mouse	500.0 mg/kg	Inactive [81]
Procyanidin B-1 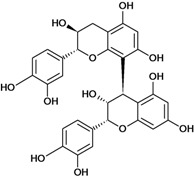	Stress-induced ulcers (water-immersion)/gastric intubation	Mouse	500.0 mg/kg	Weak activity [81]
Procyanidin B-2 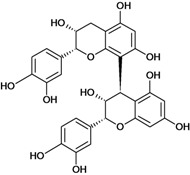	Stress-induced ulcers (water-immersion)/gastric intubation	Mouse	200.0 mg/kg	Active [81]
Procyanidin B-3 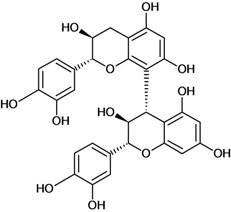	HCl/ethanol-induced stomach ulcers/intragastric	Rat	200.0 mg/kg	Inactive [79]
Procyanidin B-4 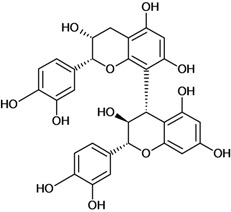	Stress-induced ulcers (water-immersion)/gastric intubation	Mouse	500.0 mg/kg	Active [81]

* Dates weren’t provided

## Conclusions

Flavonoids represent a highly diverse class of secondary metabolites with potentially beneficial effects on human health. These compounds protect the gastrointestinal mucosa from lesions produced by various experimental ulcer models and against different necrotic agents. Several mechanisms of action may be involved in this protective effect. Quercetin has an anti-secretory mechanism of action. This flavonol has antihistaminic properties, thus, decreases histamine levels, as well as preventing the release of histamine from gastric mast cells and inhibiting the gastric H+/K+ proton pump, diminishing acid gastric secretion. On the other hand chalcones, in particular those with more than one isoprenyloxyl group, possess cytoprotective effects, which increase the mucosal blood flow, stimulate the synthesis of mucosubstances in the gastric mucosa and increase PGs levels. However, the most important mechanism of action responsible for the anti-ulcer activity of flavonoids is their antioxidant properties, seen in garcinol, rutin and quercetin, which involves free radical scavenging, transition metal ions chelation, inhibition of oxidizing enzymes, increase of proteic and nonproteic antioxidants and reduction of lipid peroxidation. These effects are correlated with presence in the structures of an *o*-dihydroxy in the ring B (catechol), and additionally a 2,3 double bond in conjugation with a 4-oxo function, as well as the presence hydroxyl groups in positions 3, 5 and 7. Besides the gastroprotective activity, sofalcone (a chalcone), quercetin and naringenin (flavanones) accelerate the healing of gastric ulcers. In addition, the two first polyphenolic compounds have anti-*H. pylori* activity and may be utilized as an alternative or additive agent to the current therapy. Therefore flavonoids could have an ideal more effective and less toxic therapeutic potential for the treatment of gastrointestinal diseases, particularly for peptic ulcers.
